# Association between *IL1B* gene and cervical cancer susceptibility in Chinese Uygur Population: A Case–Control study

**DOI:** 10.1002/mgg3.779

**Published:** 2019-06-20

**Authors:** Li Wang, Wenhui Zhao, Jiajing Hong, Fanglin Niu, Jing Li, Shanshan Zhang, Tianbo Jin

**Affiliations:** ^1^ Key Laboratory of Molecular Mechanism and Intervention Research for Plateau Diseases of Tibet Autonomous Region School of Medicine, Xizang Minzu University Xianyang Shaanxi People's Republic of China; ^2^ School of Basic Medical Sciences Xizang Minzu University Xianyang Shaanxi People's Republic of China; ^3^ Department of Anesthesiology Shaanxi Provincial Cancer Hospital Xi'an Shaanxi People's Republic of China; ^4^ College of Acupuncture and Massage Changchun University of Traditional Chinese Medicine Changchun Jilin People's Republic of China; ^5^ Key Laboratory of Resource Biology and Biotechnology in Western China (Northwest University) Ministry of Education Xi'an Shaanxi People's Republic of China

**Keywords:** Case–Control study, cervical cancer, *IL‐1B* gene, single nucleotide polymorphism, susceptibility

## Abstract

**Background:**

Interleukin‐1β (IL‐1B) has been recognized as a pro‐inflammatory cytokine and associated with tumorigenesis. We aimed to evaluate the contribution of *IL‐1B* polymorphisms to the susceptibility of cervical cancer in Chinese Uygur population.

**Methods:**

Seven variants were genotyped by Agena MassARRAY platform in 267 cervical cancer patients and 302 healthy controls. Allelic, genotypic, and haplotypic association analyses adjusted for age were investigated using odds ratios (OR) and 95% confidence intervals (CI). GEPIA and UALCAN databases were used to evaluate expression and prognostic of *IL‐1B* gene in cervical cancer.

**Results:**

Our result revealed *IL‐1B* rs1143627‐AA (OR = 1.98, *p* = 0.029) and rs16944‐GG (OR = 2.01, *p* = 0.025) was associated with an increased risk of cervical cancer. Besides, we also found two protective single nucleotide polymorphisms (SNPs) rs3136558 (OR = 0.63, *p* = 0.011) and rs1143630 (OR = 0.63, *p* = 0.019). Haplotype ″TGA″ in the block (rs1143630, rs1143627, and rs16944) significantly decreased the susceptibility of cervical cancer (OR = 0.53, *p* = 0.0007). *IL‐1B* mRNA level was up‐regulated in the cervical cancer patients, which was related with poor prognosis in silico.

**Conclusions:**

For the first time, our results provide evidence on polymorphism of *IL‐1B* gene associated with cervical cancer risk in Chinese Uygur population.

## 1 INTRODUCTION

Cervical cancer is considered to be the second most common gynecologic tumor after breast cancer and one of the main leading causes of cancer‐related mortality among women worldwide. It is estimated to account for around 570,000 new cases and 311,000 deaths in 2018 global cancer statistics, this disease ranks as the fourth most frequently diagnosed cancer and the fourth leading cause of cancer death in women(Bray et al., 2018). More than 100,000 new cervical cancer cases are diagnosed in China every year, and many individuals have not yet been diagnosed (Chen et al., 2016). It is generally thought to be a multifactorial disease as the dynamic interactions between environmental exposures and host genetic background. Genetic polymorphisms are now recognized as a major cause of the disease. Previous studies have reported that many genetic variations in immune‐associated genes including tumor necrosis factor, interleukin (IL), and DNA repair genes may play a crucial role in the cervical carcinogenesis risk (Pontillo et al., 2016). Interleukins (ILs) are a group of cytokines, which are involved in the immune and inflammatory responses and are involved in the development of tumors. It is reported that several interleukin gene polymorphisms (such as *IL‐1*, *IL‐6,* and *IL‐10*) are associated with risk of cervical cancer (Guo et al., 2018; Zidi et al., 2015, 2017).

Interleukin‐1 (IL‐1) is one of the endogenous cytokine families, which is produced by monocytes, macrophages, and epithelial cells and involved in inflammatory, immunological responses, and cancer formation (Sims & Smith, 2010). *IL‐1* gene family located in locus 2q13‐21 has three genes consisting of the IL‐1 alpha (*IL‐1A*), IL‐1 beta (*IL‐1B*), and IL‐1 receptor antagonist (*IL‐1RN*). These genes encode the pro‐inflammatory cytokines IL‐1α, IL‐1β, and the anti‐inflammatory cytokine IL‐1ra, respectively (Arend, Malyak, And, & Gabay, 1998). Among it, IL‐1β is a pro‐inflammatory cytokine mainly produced by blood monocytes and tissue macrophages in both acute and chronic inflammation. Solid tumors in which IL‐1β has been shown to be up‐regulated include breast, colon, lung, and melanomas, and patients with IL‐1β producing tumors have generally bad prognoses (Lewis, Varghese, Xu, & Alexander, 2006). With respect to cervical cancer, studies have revealed that the positive association with increased IL‐1β secretion and cervical cancer risk (Magdy A. Al‐Tahhan, Etewa, & Behery, 2011). Combined with published literature, *IL‐1B* polymorphisms (rs2853550, rs1143643, rs3136558, rs1143630, rs1143627, rs16944, and rs1143623) are associated with the susceptibility of several tumors (Ban, Kim, Park, & Kwon, 2012; Xu, Ding, & Jiang, 2014).

To date, less study has been conducted for investigating the association between *IL‐1B* polymorphisms and the susceptibility to cervical cancer, and there has been no relevant reported data in Chinese Uygur populations. Therefore, a Case–Control study was carried out to evaluate the possible correlation of *IL‐1B* polymorphisms with the risk of cervical cancer among Chinese Uygur population.

## 2 MATERIAL AND METHODS

### 2.1 Study participants

This Case–Control study involving a genetically unrelated Chinese Uyghur population of 247 patients with cervical cancer and 286 healthy controls was performed. All patients were recruited from the people's Hospital of Xinjiang Uygur Autonomous Region. All included patients had recently diagnosed and histopathologically confirmed primary cervical carcinoma according to the International Federation of Gynecology and Obstetrics classification criteria. All patients were judged by two or three independent gynecologists. Patients who had any history of cancer and received either radiotherapy or chemotherapy before surgery were excluded. The controls were randomly recruited from the health checkup center of the people's Hospital of Xinjiang Uygur Autonomous Region, where they visited for an annual health examination during the same period. All of the controls were confirmed to be cervical cytology negative in the pathology department, and ascertained to be no history of cancer, infection, and free from any acute or chronic pathology. Moreover, individuals with cervical cancer family history of more than three generations were eliminated.

### 2.2 Data collection

Peripheral blood (5 ml) was collected from each subject into tubes containing ethylenediamine tetraacetic acid at the time of initial diagnosis, and stored at −20°C until further use. All subjects agreed the purpose and experimental procedures of the study, and the basic characteristics of all participants were collected with a standard epidemiological questionnaire conducted by well‐trained interviewers. Written informed consent was obtained from all of the subjects before participating. The protocol of this study was approved by the institutional Ethnics Committee of both the People's Hospital of Xinjiang Uygur Autonomous Region and Xi'an Jiaotong University, and carried out in accordance with the World Medical Association Declaration of Helsinki.

### 2.3 Single nucleotide polymorphism genotyping

Seven single nucleotide polymorphisms (SNPs) in the *IL‐1B* gene (rs2853550, rs1143643, rs3136558, rs1143630, rs1143627, rs16944, and rs1143623) were selected from previously published polymorphisms associated with cervical cancer or other cancers for further genotyping (Liu, Wang, Yu, Lei, & Wang, 2010; Peng et al., 2011). The minor allele frequency of each SNP was >5% in the CHB data from the 1000 Genomes Project. The commercially available GoldMag whole blood genomic DNA purification kit (GoldMag Co. Ltd., Xiʹan, China) was used to extract DNA from peripheral blood samples according to the manufacturerʹs protocol. DNA concentrations and qualities were evaluated with the NanoDrop 2000C (Thermo Scientific, Waltham, Massachusetts, USA). The isolated DNA was stored at −80°C until analysis. SNP genotyping was performed using the Agena MassARRAY Assay (Agena, San Diego, CA, USA) based on the standard manufacturerʹs protocol (Dai et al., 2016; Gabriel, Ziaugra, & Tabbaa, 2009). The Agena MassARRAY Assay Design 3.0 Software (San Diego, California, USA) was used to design the primers for amplification and single base extension reactions (Table 1). The data management and analysis were implemented using Agena Typer 4.0 software as previously described. For these SNPs, about 10% of the samples were randomly selected to repeat the genotyping procedure with different researchers to quality control, and the reproducibility was 100%.

**Table 1:** Primers sequence of PCR and UEP used in this study

SNP	First primer (5′‐3′)	Second primer (5′‐3′)	UEP SEQ (5′‐3′)
rs2853550	ACGTTGGATGCGAAGACTATCCTCCTCACC	ACGTTGGATGTGCAGTGCTTCAGCTGATCC	CAGCTGATCCTGTTCCA
rs1143643	ACGTTGGATGCCTCAGCATTTGGCACTAAG	ACGTTGGATGACTCCTGAGTTGTAACTGGG	GGGCCCCCAACTTTC
rs3136558	ACGTTGGATGAAGGGCTTGAAAGAATCCCG	ACGTTGGATGGATTCATCCACCTCGGCTTC	aaccCGCCTGGCCCAGAGAGGGATGA
rs1143630	ACGTTGGATGTCTTGAGTCTGCCTCTAACC	ACGTTGGATGAGATTATCCCTCTCTGAAGC	AGCTCAAGGAGGTTAAG
rs1143627	ACGTTGGATGTCTCAGCCTCCTACTTCTGC	ACGTTGGATGTTGTGCCTCGAAGAGGTTTG	gtTCCCTCGCTGTTTTTAT
rs16944	ACGTTGGATGCTGTCTGTATTGAGGGTGTG	ACGTTGGATGAGAGGCTCCTGCAATTGACA	AATTGACAGAGAGCTCC
rs1143623	ACGTTGGATGACCTATTTCCCTCGTGTCTC	ACGTTGGATGATGTGCCAGGTATCGTGCTC	tttaGTGCTCGCTCTGCATTAT

Abbreviations: SNP, single nucleotide polymorphism; UEP, unextended mini sequencing primer.

### 2.4 Bioinformatics analysis

RegulomeDB (http://regulome.stanford.edu/index) and HaploReg v4.1 (https://pubs.broadinstitute.org/mammals/haploreg/haploreg.php) were used to predict the potential functions of the candidate SNPs. The mRNA expression and prognostic significance of *IL‐1B* gene in cervical cancer were evaluated using GEPIA (Gene Expression Profiling Interactive Analysis, http://gepia.cancer-pku.cn/) and UALCAN (http://ualcan.path.uab.edu/analysis.html).

### 2.5 Data analysis

Statistical analysis was performed using SPSS software (version 19.0, SPSS Inc., Chicago, USA). Genotype frequencies in controls were calculated for departure from Hardy–Weinberg equilibrium (HWE) using a Fisher's exact test, which was performed by comparing the observed and expected genotype frequencies in controls. During the analysis, Pearson χ^2^ tests or Fisher exact test was used to compare the allele and genotype frequencies of *IL‐1B* between cervical cases and healthy controls, as appropriate. The association between the allelic frequencies of *IL‐1B* and the risk of cervical cancer was estimated by calculating odds ratios (ORs), 95% confidence intervals (95% CIs), and their corresponding *p*‐values. Subsequently, multiple genetic model analyses (codominant, dominant, recessive, log‐additive) were applied using SNPstats (http://bioinfo.iconcologia.net/snpstats/start.htm) to estimate the main effects of SNPs. For each polymorphism, ORs and 95% CIs were used for unconditional logistic regression analysis with adjustment for age. Finally, the pairwise linkage disequilibrium (LD) and haplotype construction was performed using Haploview software package (version 4.2), and haplotype association analyses were conducted with SHEsis software. All *p*‐values were two‐sided, and *p* < 0.05 was considered as statistically significant differences for all the analyses.

## 3 RESULTS

### 3.1 Study participants

In this Case–Control study, a total of 533 participants were enrolled including 247 cervical cases (mean age at 54.55 ± 10.31 years) and 286 healthy controls (mean age at 50.82 ± 15.18 years).

### 3.2 In silico analysis predicted the function of the selected SNPs

To evaluate the possible function of the selected SNPs, we conducted in silico analysis using Regulome DB Score and HaploReg. Table 2 showed the basic information and the potential function of the selected SNPs. Regulome DB database allows assessing functional effects of SNPs in non‐coding and intergenic regions using known and predicted regulatory elements. The RegulomeDB scores refer to the data available for each individual SNP, with lower scores represented the more important the function. By HaploReg annotation, we found that the selected SNPs were associated with regulation of promoter and/or enhancer histone, DNase, proteins bound, motifs changed, selected eQTL hits.

**Table 2:** In silico analysis for SNPs function annotation

SNP	Chr: Position	Role	Allele^a^	Regulome DB Score	HaploReg
rs2853550	2:113587121	Downstream	A/G	3a	Enhancer histone, DNase, Proteins bound, Motifs changed
rs1143643	2:113588302	Intron	T/C	6	Enhancer histone, DNase, Motifs changed, Selected eQTL hits
rs3136558	2:113591275	Intron	G/A	5	Promoter and Enhancer histone, DNase, Motifs changed
rs1143630	2:113591655	Intron	T/G	/b	Promoter and Enhancer histone, DNase, Motifs changed
rs1143627	2:113,594,387	Promoter	A/G	1b	Promoter and Enhancer histone, DNase, Proteins bound, Motifs changed, Selected eQTL hits
rs16944	2:113594867	Promoter	G/A	1f	Promoter and Enhancer histone, DNase, Motifs changed, Selected eQTL hits
rs1143623	2:113595829	Promoter	G/C	/	Promoter and Enhancer histone, DNase, Motifs changed, Selected eQTL hits

Abbreviation: SNP, single nucleotide polymorphism.

a Effect allele/reference allele.

b /: No data.

### 3.3 Correlation between *IL‐1B* SNPs and risk of cervical cancer

Seven SNPs in the *IL‐1B* gene were successfully genotyped for further analysis in patients and healthy controls. The genotype distribution for all of the tested SNPs was in accordance with HWE among the control participants (*p* > 0.05). The call rate of SNPs was above 99.2% in case and controls. The minor allele of each SNP as a risk factor was compared to the wild‐type (major) allele. The differences in the frequency distribution of alleles between cases and controls were compared using Pearson χ^2^ test and ORs. We found that four significant SNPs (rs3136558, rs1143630, rs1143627, and rs16944) were associated with the susceptibility of cervical cancer in allele model, in which two SNPs significantly decreased cervical cancer risk (rs3136558, OR = 0.77, 95% CI: 0.60–0.99, *p* = 0.045; rs1143630, OR = 0.60, 95% CI: 0.43–0.84, *p* = 0.003) and two SNPs increased the risk (rs1143627, OR = 1.35, 95% CI: 1.06–1.72, *p* = 0.015; rs16944, OR = 1.35, 95% CI: 1.06–1.72, *p* = 0.014), as shown in Table 3. However, no evidence of the association was observed between rs2853550, rs1143643, and rs1143623 polymorphisms and risk of cervical cancer.

**Table 3:** Allelic model analysis and HWE analysis about *IL1B* candidate SNPs

SNP ID	Alleles (minor/major)	Frequency (MAF)		*p*‐Value for HWE	Call rate (%)	OR (95% CI)	*p*
		Case	Control				
rs2853550	A/G	0.101	0.136	0.075	100%	0.71 (0.49–1.04)	0.078
rs1143643	T/C	0.415	0.379	0.530	100%	1.16 (0.91–1.49)	0.231
rs3136558	G/A	0.325	0.384	0.614	99.6%	**0.77 (0.60–0.99)**	0.045*
rs1143630	T/G	0.128	0.196	0.061	100%	**0.60 (0.43–0.84)**	0.003*
rs1143627	A/G	0.547	0.472	0.721	100%	**1.35 (1.06–1.72)**	0.015*
rs16944	G/A	0.551	0.475	0.812	100%	**1.35 (1.06–1.72)**	0.014*
rs1143623	G/C	0.367	0.394	0.264	99.2%	0.89 (0.70–1.15)	0.381

Abbreviations: HWE, Hardy–Weinberg equilibrium; MAF, minor allele frequency; SNP, single nucleotide polymorphism.

*p* values were calculated from Chi‐square/Fisher's exact. *p* < 0.05 indicates statistical significance. Bold indicates statistical significance.

### 3.4 Genetic models analysis the association between *IL‐1B* and cervical cancer risk

Furthermore, multiple genetic models (dominant, recessive, additive, and co‐dominant models) were applied for analyzing the association between the SNPs in the *IL‐1B* gene and cervical cancer risk by unconditional logistic regression analysis adjusted for age. It is noteworthy that four specific SNPs associated with cervical cancer risk, as displayed in Table 4. Our results identified that the rs3136558 decreased the risk of cervical cancer in co‐dominant model (OR = 0.60, CI: 0.41–0.87, *p* = 0.028) and in dominant model (OR = 0.63, CI: 0.44–0.90, *p* = 0.011). Rs1143630 was also associated with the reduced cervical cancer risk by dominant model analyses (OR = 0.63, CI: 0.43–0.93, *p* = 0.019) and log‐additive model analyses (OR = 0.57, CI: 0.40–0.81, *p* = 0.001). Conversely, two SNPs (rs1143627 and rs16944) that increased cervical cancer risk were observed on the basis of codominant model (rs1143627, OR = 1.98, CI: 1.19–3.30, *p* = 0.029; rs16944 OR = 2.01, CI: 1.21–3.34, *p* = 0.025), the dominant model (rs1143627, OR = 1.61, CI: 1.06–2.45, *p* = 0.024; rs16944, OR = 1.65, CI: 1.08–2.51, *p* = 0.019), recessive model (rs1143627, OR = 1.52, CI: 1.02–2.28, *p* = 0.041; rs16944, OR = 1.52, CI: 1.02–2.26, *p* = 0.041), and the log‐additive model (rs1143627, OR = 1.41, CI: 1.09–1.81, *p* = 0.008; rs16944, OR = 1.41, CI: 1.10–1.82, *p* = 0.007).

**Table 4:** Relationship between *IL1B* gene polymorphisms and risk of cervical cancer under multiple models of inheritance

SNP ID	Model	Genotype	Control	Case	Crude analysis		Adjusted by age and gender	
					OR (95%CI)	*p*‐Value	OR (95%CI)	*p*‐Value
rs2853550	Codominant	G/G	217 (75.9%)	203 (82.2%)	1.00	0.200	1.00	0.210
		A/G	60 (21.0%)	38 (15.4%)	0.68 (0.43–1.06)		0.69 (0.44–1.08)	
		A/A	9 (3.1%)	6 (2.4%)	0.71 (0.25–2.04)		0.65 (0.22–1.87)	
	Dominant	G/G	217 (75.9%)	203 (82.2%)	1.00	0.074	1.00	0.077
		A/G‐A/A	69 (24.1%)	44 (17.8%)	0.68 (0.45–1.04)		0.68 (0.44–1.05)	
	Recessive	G/G‐A/G	277 (96.8%)	241 (97.6%)	1.00	0.620	1.00	0.490
		A/A	9 (3.1%)	6 (2.4%)	0.77 (0.27–2.18)		0.69 (0.24–2.00)	
	Log‐additive	—	—	—	0.74 (0.52–1.06)	0.096	0.73 (0.51–1.05)	0.087
rs1143643	Codominant	C/C	107 (37.5%)	82 (33.2%)	1.00	0.470	1.00	0.520
		C/T	140 (49.1%)	125 (50.6%)	1.17 (0.80–1.70)		1.17 (0.80–1.71)	
		T/T	38 (13.3%)	40 (16.2%)	1.37 (0.81–2.33)		1.34 (0.79–2.30)	
	Dominant	C/C	107 (37.5%)	82 (33.2%)	1.00	0.300	1.00	0.310
		C/T‐T/T	178 (62.5%)	165 (66.8%)	1.21 (0.85–1.73)		1.21 (0.84–1.74)	
	Recessive	C/C‐C/T	247 (86.7%)	207 (83.8%)	1.00	0.350	1.00	0.410
		T/T	38 (13.3%)	40 (16.2%)	1.26 (0.78–2.03)		1.23 (0.75–2.00)	
	Log‐additive	—	—	—	1.17 (0.91–1.51)	0.220	1.16 (0.90–1.50)	0.250
rs3136558	Codominant	A/A	104 (37.0%)	118 (48.0%)	1.00	0.033*	1.00	0.028*
		G/A	138 (49.1%)	96 (39.0%)	**0.61 (0.42–0.89)**		**0.60 (0.41–0.87)**	
		G/G	39 (13.9%)	32 (13.0%)	0.72 (0.42–1.24)		0.76 (0.44–1.31)	
	Dominant	A/A	104 (37.0%)	118 (48.0%)	1.00	0.011*	1.00	0.011*
		G/A‐G/G	177 (63.0%)	128 (52.0%)	**0.64 (0.45–0.90)**		**0.63 (0.44–0.90)**	
	Recessive	A/A‐G/A	242 (86.1%)	214 (87.0%)	1.00	0.770	1.00	0.960
		G/G	39 (13.9%)	32 (13.0%)	0.93 (0.56–1.53)		0.99 (0.59–1.64)	
	Log‐additive	—	—	—	0.78 (0.60–1.00)	0.049	0.79 (0.61–1.02)	0.064
rs1143630	Codominant	G/G	190 (66.4%)	184 (74.5%)	1.00	**/**	1.00	**/**
		G/T	80 (28.0%)	63 (25.5%)	0.81 (0.55–1.20)		0.76 (0.51–1.13)	
		T/T	16 (5.6%)	**0 (0.00%)**	**/**		**/**	
	Dominant	G/G	190 (66.4%)	184 (74.5%)	1.00	0.042*	1.00	0.019*
		G/T‐T/T	96 (33.6%)	63 (25.5%)	**0.68 (0.46–0.99)**		**0.63 (0.43–0.93)**	
	Recessive	G/G‐G/T	270 (94.4%)	247 (100.0%)	1.00	**/**	1.00	**/**
		T/T	16 (5.6%)	0 (0.0%)	**/**		**/**	
	Log‐additive	—	—	—	**0.60 (0.43–0.85)**	0.003*	**0.57 (0.40–0.81)**	0.001*
rs1143627	Codominant	G/G	77 (27.2%)	47 (19.0%)	1.00	0.042*	1.00	0.029*
		A/G	145 (51.2%)	130 (52.6%)	1.47 (0.95–2.27)		1.46 (0.94–2.27)	
		A/A	61 (21.6%)	70 (28.4%)	**1.88 (1.14–3.10)**		**1.98 (1.19–3.30)**	
	Dominant	G/G	77 (27.2%)	47 (19.0%)	1.00	0.026*	1.00	0.024*
		A/G‐A/A	206 (72.8%)	200 (81.0%)	**1.59 (1.05–2.40)**		**1.61 (1.06–2.45)**	
	Recessive	G/G‐A/G	222 (78.5%)	177 (71.7%)	1.00	0.071	1.00	0.041*
		A/A	61 (21.6%)	70 (28.3%)	1.44 (0.97–2.14)		**1.52 (1.02–2.28)**	
	Log‐additive	—	—	—	**1.37 (1.07–1.76)**	0.013*	**1.41 (1.09–1.81)**	0.008*
rs16944	Codominant	A/A	77 (27.0%)	46 (18.6%)	1.00	0.039*	1.00	0.025*
		G/A	145 (50.9%)	130 (52.6%)	1.50 (0.97–2.32)		1.50 (0.96–2.33)	
		G/G	63 (22.1%)	71 (28.7%)	**1.89 (1.15–3.11)**		**2.01 (1.21–3.34)**	
	Dominant	A/A	77 (27.0%)	46 (18.6%)	1.00	0.021*	1.00	0.019*
		G/A‐G/G	208 (73.0%)	201 (81.4%)	**1.62 (1.07–2.45)**		**1.65 (1.08–2.51)**	
	Recessive	A/A‐G/A	222 (77.9%)	176 (71.3%)	1.00	0.079	1.00	0.041*
		G/G	63 (22.1%)	71 (28.7%)	1.42 (0.96–2.11)		**1.52 (1.02–2.26)**	
	Log‐additive	—	—	—	**1.37 (1.07–1.75)**	0.013*	**1.41 (1.10–1.82)**	0.007*
rs1143623	Codominant	C/C	99 (35.1%)	91 (37.1%)	1.00	0.490	1.00	0.490
		C/G	144 (51.1%)	128 (52.2%)	0.92 (0.63–1.35)		0.92 (0.63–1.35)	
		G/G	39 (13.8%)	26 (10.6%)	0.70 (0.39–1.26)		0.70 (0.39–1.26)	
	Dominant	C/C	99 (35.1%)	91 (37.1%)	1.00	0.470	1.00	0.470
		C/G‐G/G	183 (64.9%)	154 (62.9%)	0.88 (0.61–1.26)		0.88 (0.61–1.26)	
	Recessive	C/C‐C/G	243 (86.2%)	219 (89.4%)	1.00	0.260	1.00	0.260
		G/G	39 (13.8%)	26 (10.6%)	0.74 (0.43–1.26)		0.74 (0.43–1.26)	
	Log‐additive	—	—	—	0.88 (0.68–1.15)	0.360	0.86 (0.66–1.13)	0.280

Abbreviations: OR, Odd ratio; 95% CI, 95% confidence interval.

*p* < 0.05 indicates statistical significance.

“*” means the data are statistically significant. Bold indicates statistical significance.

“/” means no data.

### 3.5 Haplotype analysis

**Figure 1:**
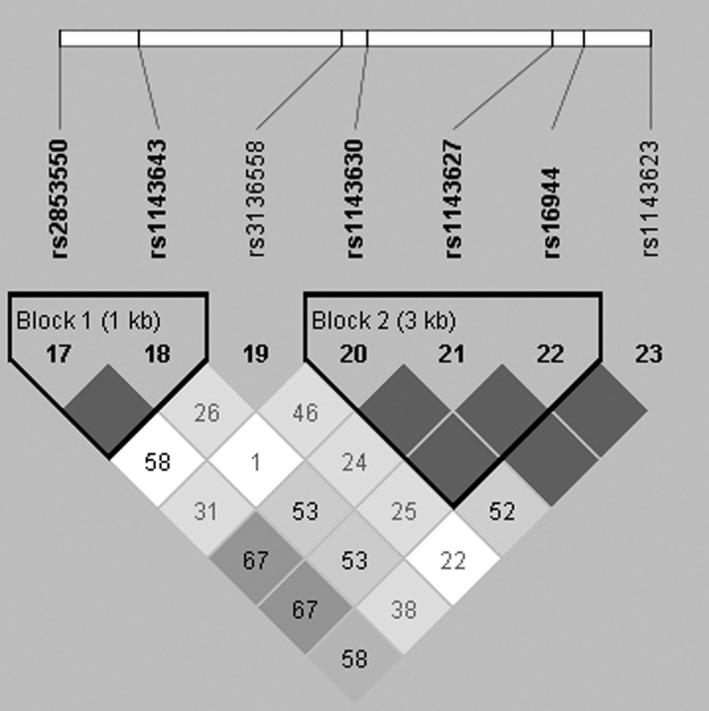
Haplotype block map for the SNPs of the *IL‐1B* gene. Block 1 includes rs2853550 and rs1143643; Block 2 includes rs1143630, rs1143627 and rs16944. The LD between two SNPs is standardized D′. LD, linkage disequilibrium; SNP, single nucleotide polymorphism.

Subsequently, haplotype analysis was used to explore the association of *IL‐1B* polymorphisms with cervical cancer susceptibility. LD analysis demonstrated the existence of two blocks (Block 1: rs2853550 and rs1143643; Block 2: rs1143630, rs1143627, and rs16944) in the *IL‐1B* gene (Figure 1). Furthermore, haplotype TGA was found to significantly decrease the risk of cervical cancer by Pearson χ^2 ^tests (*p* = 0.003) and under unconditional logistic regression analysis adjusted for age (OR = 0.53, 95% CI: 0.37–0.76, *p* = 0.0007) (Table 5).

**Table 5:** *IL1B* haplotype frequencies and the association with cervical cancer in case and control subjects

	Haplotype	Freq (case)	Freq (control)	*χ* ^2^	*P* value	Crude analysis		Adjusted by age	
						OR (95% CI)	*p*	OR (95% CI)	*p*
Block 1	GC	0.484	0.484	0.00	0.984	1.00		1.00	
	GT	0.415	0.379	1.42	0.234	1.10 (0.84–1.44)	0.480	1.09 (0.83–1.43)	0.540
	AC	0.101	0.136	3.10	0.078	0.77 (0.53–1.13)	0.180	0.76 (0.52–1.12)	0.160
Block 2	GAG	0.547	0.474	5.57	0.018	1.00		1.00	
	GGA	0.322	0.328	0.05	0.823	0.84 (0.64–1.11)	0.220	0.83 (0.62–1.10)	0.190
	TGA	0.128	0.196	9.01	0.003	**0.56 (0.40–0.81)**	0.002*	**0.53 (0.37–0.76)**	0.0007*

Note

Block 1, rs2853550 and rs1143643; Block 2, rs1143630, rs1143627, and rs16944.

Abbreviations: OR, Odd ratio; 95% CI, 95% confidence interval.

*p* < 0.05 indicates statistical significance. Bold indicates statistical significance.

“*” means the data are statistically significant.

### 3.6 Bioinformatics analysis of *IL‐1B* expression and prognosis

**Figure 2:**
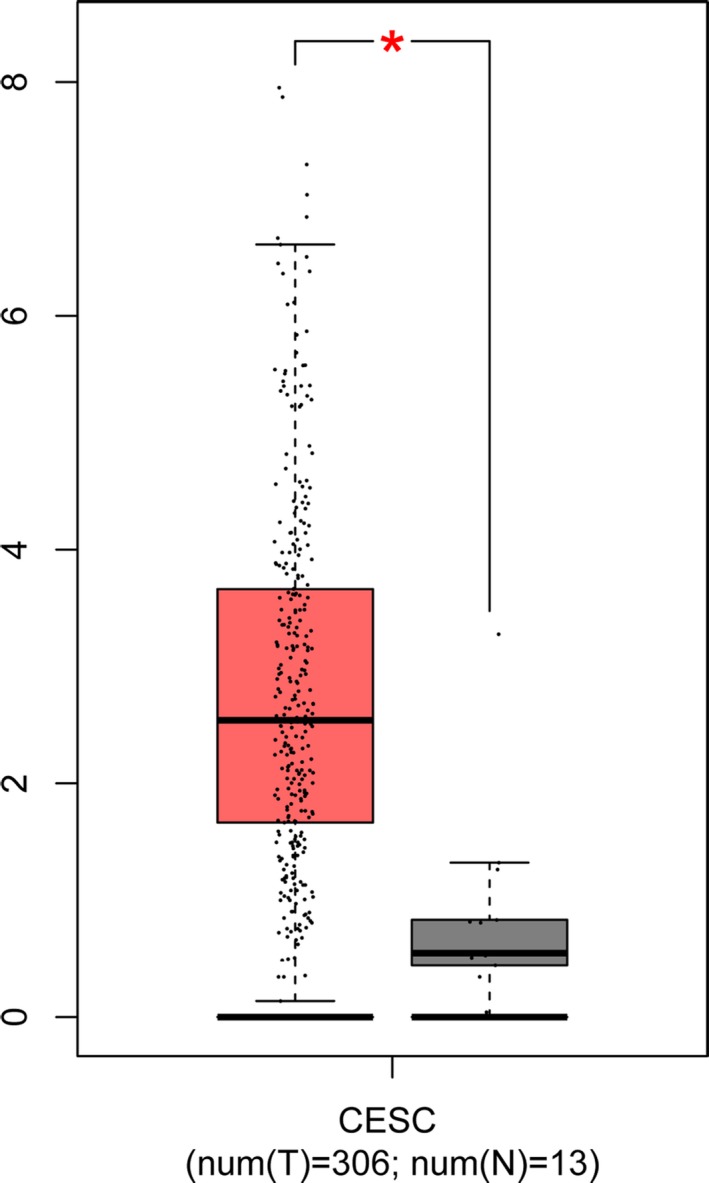
Differential expression of *IL‐1B* in cervical tumor tissues and normal tissues. Each bar represents the average level of *IL‐1B* expression. Error bars represent the standard deviation of the mean value. Data were extracted from the GEPIA database (http://gepia.cancer-pku.cn/). *indicates statistical significance (*p* < 0.01)

**Figure 3:**
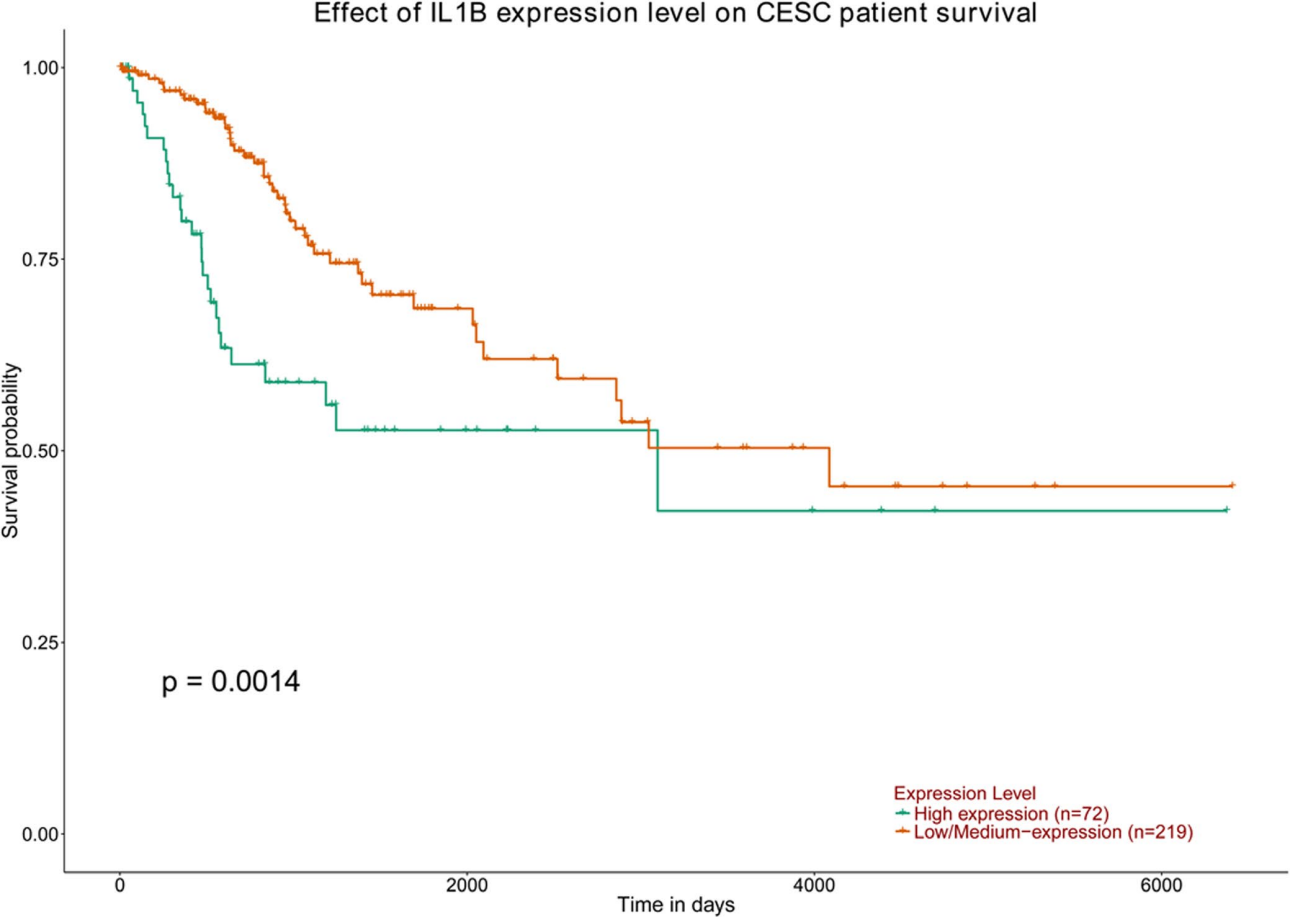
*IL‐1B* high expression is associated with poor survival in cervical cancer. Kaplan–Meier plots of overall survival: comparison of patients with high versus low/Medium expression of *IL‐1B* in cervical cancer patients. The Kaplan–Meier plots were generated by UALCAN (http://ualcan.path.uab.edu/analysis.html).

We investigated mRNA levels of *IL‐1B* gene and the expression with prognosis in cervical cancer using GEPIA and UALCAN databases. As shown in Figure 2, *IL‐1B* mRNA expression was significantly increased in cervical cancer tissues compared with that in normal tissues (*p* < 0.01), suggesting an important role of *IL‐1B* in the development of cervical cancer. Besides, UALCAN database assessed the association between the expression of *IL‐1B* gene and the prognosis of cervical cancer. Survival curves were plotted for 391 cervical cancer cases (Figure 3) and the result found that the high expression level of *IL‐1B* significantly increased the death risk of cervical cancer (*p* = 0.0014).

## 4 DISCUSSION

In this Case–Control study, seven SNPs (rs2853550, rs1143643, rs3136558, rs1143630, rs1143627, rs16944, and rs1143623) were successfully genotyped and found some evidence of association with four SNPs associated with cervical cancer risk. The results suggested that rs3136558‐G and rs1143630‐T were protective alleles, and rs1143627‐A and rs16944‐G significantly increased the susceptibility to cervical cancer. Furthermore haplotype association analysis revealed that TGA (rs1143630, rs1143627, and rs16944) trends to decrease cervical cancer risk. To the best of our knowledge, this is the first study that has investigated these SNPs in the *IL1B* gene are associated with cervical cancer risk in Chinese Uygur female.

In recent years, a considerable evidence has supported the concept that inflammation plays important roles in the progression and susceptibility of many pathological disorders or diseases, especially tumor (Jin et al., 2016, 2013; Li et al., 2012). Furthermore, inflammation‐related genetic factors could be important in the pathogenesis of cervical cancer through alteration of the inflammatory state or interaction with environmental factors (Wu, Hu, Chen, & Xie, 2014). IL‐1β, as a pro‐inflammatory cytokine, is often associated with tumor invasion and metastasis closely. Several studies have demonstrated that certain genotypes of the *IL‐1B* gene may be related to the susceptibility of cervical carcinoma, and found IL‐1β has higher level of plasma in cervical cancer cases (Al‐Tahhan et al., 2011). According to GEPIA and UALCAN databases, *IL‐1B* overexpression was observed in cancer tissues and correlated with a shorter survival of cervical cancer patients.

*IL‐1B* A‐31G (rs1143627) and G‐511A (rs16944), located in the promoter region, are in complete LD, which may influence the gene and protein expressions of IL‐1β. Especially, SNP rs1143627 situated in a TATA‐box motif markedly influence the transcription activity of *IL‐1B* gene (Elomar et al., 2000). To date, many epidemiological studies have investigated the association of *IL‐1B* rs1143627 and rs16944 and the susceptibility of various cancers, referring gastric, breast, lung, and prostate cancer, even cervix (Liu et al., 2010; Pérez‐Ramírez et al., 2016). Rs16944 AA genotype of the *IL‐1B* gene was found to be significantly associated with higher cervical cancer risk (OR = 2.16, *p* = 0.028) in the Egyptian population (Altahhan et al., 2011). Similar, persistent HPV16/18 infection in Indian population with the A‐allele (rs16944) of *IL‐1B* is associated with development of cervical carcinoma (Dutta et al., 2015). However, among the notable findings in our study was the observation that *IL‐1B* rs16944‐G was associated with increased risk of cervical cancer, which is inconsistent with previous results. The dissimilarity in these reports may result from allelic heterogeneity among the different ethnic groups, the complexity of gene and environment interaction or the small sample size. In addition, we also found that AA genotype of rs1143627 increased cervical cancer risk. Rs1143627‐A has been found to have significantly higher IL‐1β expression, resulting in the developing cancer (H. Chen et al., 2006). This is in accordance with our results. Moreover, A allele of rs1143627 was significantly associated with breast cancer as a protective effect and gastric cancer as a risk factor, but the *IL‐1B* rs1143627 polymorphism was no influence on non‐small cell lung cancer clinical outcomes (Chang et al., 2005; Ito et al., 2002; Perez‐Ramirez et al., 2017). These results suggested that *IL‐1B* ‐31A/G polymorphism may play different roles in the pathogenesis of different cancer types and the function of the *IL‐1B* rs1143627 remains to be further investigated.

Very few studies have analyzed the SNPs rs3136558 and rs1143630 to now. Only three previous studies have analyzed rs3136558 polymorphism, which reported *IL‐1B* (rs3136558) was significantly associated with new‐onset diabetes after transplantation and papillary thyroid carcinoma (Ban et al., 2012; Kim et al., 2012), and a few studies have indicated the associations of rs1143630 with preeclampsia and papillary thyroid carcinoma (Ban et al., 2012; Galvão et al., 2016). However, we failed to find some research that referred to associations between the SNPs rs3136558 and rs1143630 and risk of cervical cancer, even related cancer. In our study, the result revealed that rs3136558 and rs1143630 of *IL‐1B* may be protective loci for development of cervical cancer. Rs3136558 polymorphism of *IL‐1B* in the co‐dominant and dominant models showed that *IL1B* rs3136558 GA heterozygotes had a decreased risk of cervical cancer. Moreover, rs1143630 in dominant and log‐additive models revealed a weak protective effect against the development of cervical cancer. However, these results could have been due to our relatively small sample size and need large samples to further verification.

However, several limitations of our study should not be ignored. First, *IL‐1B* expression data and survival data were from the database, and we did not verify its validity by this study. Therefore, further functional studies are required to elucidate the associations and clarify the precise mechanisms. Second, there is the unavailability of complete clinical information such as histological subtypes and the absence of some environmental exposures factors such as HPV, smoking status, which should be evaluated in the future. Despite the limitations mentioned above, the results of this study provided scientific evidence of *IL‐1B* gene with cervical cancer in the future studies.

## 5 CONCLUSIONS

In conclusion, this is an exploratory study to examine putatively functional genetic variants of the *IL‐1B* gene with cervical cancer risk in Chinese Uygur population. Our study provides accumulating evidence for the association between *IL‐1B* gene polymorphism and the susceptibility of cervical cancer. Our findings may add new insight into the pro‐inflammatory cytokines, inflammation, and the etiology of cervical cancer. However, these results need to be taken with caution and further studies with a larger sample size and different populations should be conducted to confirm our results.

## CONFLICT OF INTEREST

The authors declare no conflict of interest.

## COMPLIANCE WITH ETHICAL STANDARDS

### Ethical approval

The protocol of this study was approved by the institutional Ethnics Committee of both the People's Hospital of Xinjiang Uygur Autonomous Region and Northwest University, and carried out in accordance with the World Medical Association Declaration of Helsinki.

### Informed consent

Written informed consent was obtained from all of the subjects before participating.
